# Septic Aspiration Pneumonia After Laparoscopic Adjustable Gastric Banding for Morbid Obesity

**DOI:** 10.7759/cureus.25074

**Published:** 2022-05-17

**Authors:** Tharini M Gara, Adam B Coleman, Donald P Roten, James M Rhinewalt

**Affiliations:** 1 General Surgery, Liberty University College of Osteopathic Medicine, Lynchburg, USA; 2 Internal Medicine, Liberty University College of Osteopathic Medicine, Lynchburg, USA; 3 General Surgery, Baptist Memorial Union County Hospital, New Albany, USA; 4 Internal Medicine, Baptist Memorial Union County Hospital, New Albany, USA

**Keywords:** esophageal disorder, aspiration pneumonia, adjustable gastric band complications, obesity treatment, lagb

## Abstract

The laparoscopic adjustable gastric banding (LAGB) surgery is a minimally invasive procedure performed to help with weight loss in patients with advanced obesity with a body mass index (BMI) of >40 kg/m² or above 35 kg/m² with comorbid obesity-related health conditions (hypertension, type two diabetes mellitus, obstructive sleep apnea, etc). Although this surgery is effective for weight loss, it is imperative that close follow-up and aftercare are conducted in order to circumvent severe and rare esophageal and pulmonary complications. We report a case of systemic pulmonary and esophageal complications associated with LAGB that required immediate medical and surgical intervention in a female patient. She underwent her surgery in Mexico, and she did not receive any follow-up care for 12 years, which seemingly led to this preventable situation.

## Introduction

The obesity epidemic has plagued the United States for decades and has been steadily increasing since 1999. In 2018, the prevalence of obesity was 42.4% and has since continued to climb. For individuals suffering from this condition, after failed lifestyle modifications and psychological evaluation, medical intervention including the laparoscopic adjustable gastric banding (LAGB) is an option [1-3]. Patients who are non-compliant with lifestyle modifications and aftercare as advised by physicians are at increased risk of developing complications and experiencing a decreased quality of life due to their inability to lose weight [4]. The best long-term benefits from this operation are associated with close follow-up to reduce the severity of common complications such as band erosion/slippage and rarer complications including pulmonary and esophageal pathology.

## Case presentation

The patient was a 67-year-old female who initially presented with generalized weakness, hypotension, respiratory distress, and subjective fevers. She also reported symptoms of gastric reflux and nausea consistently over the preceding months. Three days prior to her current admission, she had presented to the emergency department due to weakness, nausea, vomiting, abdominal pain, and myalgias. Her workup of an electrocardiogram (EKG), chest X-ray, complete blood count (CBC), influenza A/B antigen, and lactic acid had been unremarkable; she had been subsequently sent home with suspected early signs of a viral syndrome and instructed to return if the symptoms worsened.

The patient had a history of congestive heart failure and was taking 6.25 mg of carvedilol twice daily. Additionally, she had a history of type 2 diabetes mellitus (T2DM), stage 3b chronic kidney disease (CKD), chronic back pain, hyperlipidemia, and hypertension. Of note, she had severe obstructive sleep apnea but had been non-compliant with the use of her continuous positive airway pressure (CPAP) machine. 

Twelve years prior, the patient had undergone gastric surgery in Mexico. She had no bariatric follow-up and reported experiencing difficulty in enrolling for longitudinal care by either GI or bariatric surgeon postoperatively. She reported that she was disappointed with the amount of weight she had lost following her procedure, and she admitted to following an unhealthy diet. She had notably gone to the emergency department a year ago for esophageal spams that would present with recurrent squeezing chest pain occurring immediately after swallowing. She had been discharged from the hospital and placed on pantoprazole. At that time, she had been instructed to follow up with a general surgeon to have an esophagogastroduodenoscopy (EGD), which she had not undergone.

Her vital signs were as follows: blood pressure (BP) of 107/54 mmHg, a pulse of 125 beats per minute, temperature of 97.9 ℉, respiratory rate of 20 breaths per minute, saturation of peripheral oxygen (SpO_2_) at 90% on room air, and body mass index (BMI) of 34.58 kg/m^2^. Her physical exam demonstrated an ill-appearing obese female with rhonchi and rales in the right lung field, as well as noticeable difficulty in breathing. Her lab findings are reported in Table 1.

**Table 1:** Lab values of the patient compared to normal reference ranges

Variables	Patient value	Normal range
Hemoglobin (Hb)	9.2 g/dL	12.0–16.0 g/dL
C-reactive protein (CRP)	30 mg/dL	0.0–0.9 mg/dL
Erythrocyte sedimentation rate (ESR)	112 mm/hr	3.0–30.0 mm/hr
White blood cell (WBC)	8.7 K/uL	5.0–10.0 K/uL

**Figure 1:**
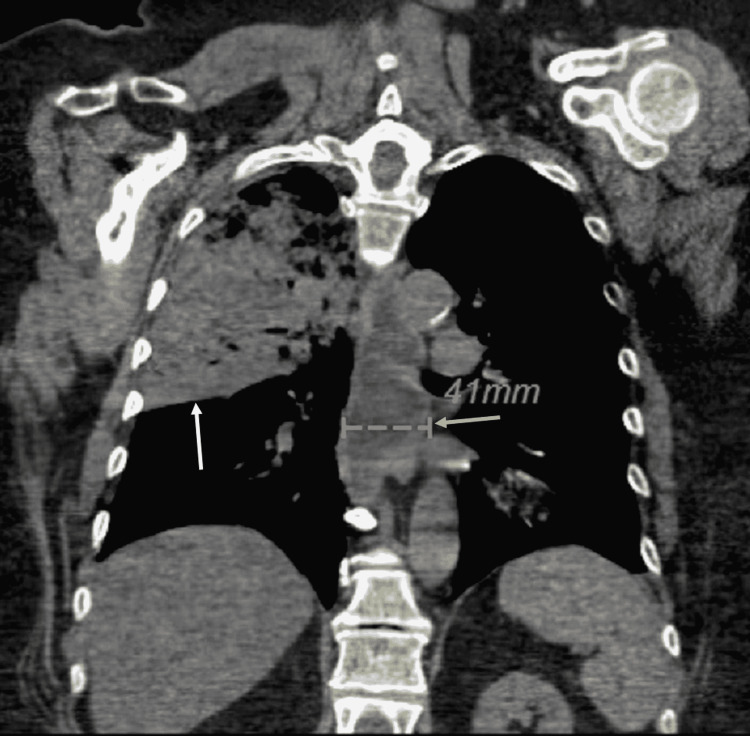
Right upper lobe and right middle lobe aspiration pneumonia marked by the white arrow. This image demonstrates the marked distal esophageal dilation (yellow arrow)

**Figure 2:**
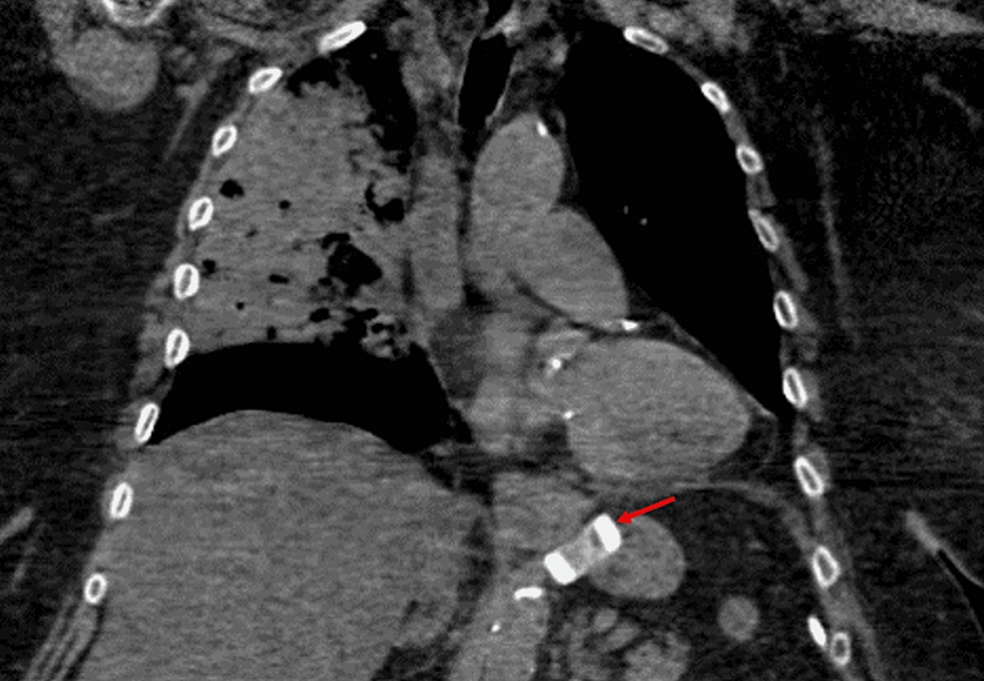
Gastric Lap Band visualized in place just distal to the gastroesophageal junction (red arrow)

A CT chest without contrast (Figure 1) demonstrated large airspace disease consolidation with air bronchograms within the right upper and right middle lobe concerning for aspiration pneumonia. The CT also demonstrated a distended fluid-filled esophagus compatible with underlying gastroesophageal reflux disease (GERD). This imaging also demonstrated her gastric Lap-Band in place (Figure 2). She had an echocardiogram, which demonstrated diastolic dysfunction and an ejection fraction of 60-65%.

The patient was admitted to the medical service unit at her local hospital for the treatment of severe aspiration pneumonia with sepsis. She was started on intravenous (IV) Zosyn. This facility was a community hospital without a bariatric program. The general surgeon on call was consulted and he percutaneously deflated her laparoscopic adjustable band. She immediately felt relief in terms of her abdominal pain. Due to the severity of her aspiration, pulmonology was consulted, and they performed a bronchoscopy, which found and removed mucous plugs throughout the tracheobronchial tree. She experienced post-procedure wheezing, which responded well to a single dose of IV steroids and nebulizer treatment and subsequently resolved. Bedside spirometry demonstrated severe obstructive lung disease, and she was started on fluticasone/umeclidinium/vilanterol (Trelegy).

She responded well to antibiotics, loosening of the band, and bronchoscopy, and was subsequently discharged after a four-day hospital stay.

## Discussion

LAGB is the third most common bariatric operation in the United States, and its associated mortality rates are the lowest compared to other common bariatric operations [5]. However, gastric banding is associated with many short- and long-term complications and requires revisional procedures in up to 40% of patients due to these complications [6]. Studies show that LAGB can lead to the loss of excess weight of 60-70% within the first three years with low complication rates, provided there is close follow-up including consultations with nutritionists and adjusting the fill of the band. Common complications of the LAGB procedure are gastric dilatation, band erosion/slippage, stomal obstruction, port tube leakage, port infection, removal of the band, and revision to gastric bypass [7]. Stomal obstruction more commonly occurs in the postoperative period of the gastric-banding procedure and is usually caused by incorrect sizing of the band over a thick gastroesophageal junction (GEJ), or improper incorporation of the gastric fundus into the band, which causes compression of the GEJ [8].

When stomal obstruction occurs in the late setting, it is usually due to esophageal or gastric pouch dilation. Esophageal dilatation often occurs in patients who do not comply with dietary restrictions. This is reversible if detected quickly and the band is properly deflated. If not managed appropriately, the esophagus can become atonic, and the esophageal dilation may become irreversible [8]. As for the patient in this case report, it is unclear whether or not her esophageal dilation is reversible or not. Pulmonary and esophageal complications such as aspiration pneumonia, esophageal dilatation, spasms, dysmotility, and increased lower esophageal sphincter pressure/duration of peristaltic wave are reported far less than other complications [9]. Obesity has a strong relation to esophageal diseases such as dysmotility and GERD, which can exacerbate complications seen in non-compliant LAGB patients, which is a feared outcome [10]. A study that investigated LAGB postoperative complications in 749 participants found a 0.3% incidence of esophageal complications (dilatation/dysmotility) and 0.8% prevalence of aspiration pneumonia [7,11]. Data concerning pulmonary complications of the LAGB surgery is scant, with one study reporting a patient with pulmonary cavitation and two patients with bronchiectasis. In light of this, physicians should be mindful of the long-term effects on a patient's pulmonary health when considering LAGB surgery. Clinical symptoms of respiratory, esophagogastric, or systemic origin such as nocturnal cough, regurgitation, and fever, should warrant further imaging such as barium radiography (esophagram/chest X-ray) if the patient has a history of undergoing LAGB surgery [12,13].

A meta-analysis of LAGB surgery data showed that 54% of patients continue with follow-up care, which leaves 46% of LAGB patients without aftercare and hence vulnerable to the risks of complications not addressed in a timely manner. Another study noted that 37% who did not follow up failed to lose weight and had a higher incidence of complications [4,14]. Additionally, studies from the United States have demonstrated disparities in access to bariatric surgery among patients residing in rural areas, patients of lower socioeconomic status, racial/ethnic minorities, and those with non-private insurance [15].

Regarding this patient, a combination of patient non-compliance and the lack of proper transfer of care from the patient's original bariatric program seemed to be the root of her problem. To prevent these serious complications, she should have had close follow-up after surgery, including annual esophagram, counseling regarding healthy lifestyle choices, and band modification (tightening/loosening) [2]. This patient, who was of lower socioeconomic status and resided in a rural area, elected to have the procedure in Mexico, which limited her access to appropriate bariatric follow-up care. This led to negative effects on her health and quality of life.

## Conclusions

LAGB is a safe and effective option for patients with morbid obesity who adhere to proper follow-up care. Some of the benefits of this procedure include weight loss, a decrease in comorbid conditions such as type 2 diabetes and GERD, and overall better quality of life. This operation is associated with low risk in the immediate postoperative period, though there are well-known long-term complications such as band erosion/slippage, port problems, and failure to lose excess weight. Clinicians should also be aware that life-threatening late pulmonary complications can occur. Providers caring for patients with LAGB or any bariatric surgical history should gather information on outpatient compliance levels and whether there has been appropriate follow-up through a bariatric provider. Arranging proper follow-up for these patients and encouraging compliance should significantly reduce long-term complications and improve weight loss.
